# Supplementary material for: Parental competition for the regulators of chromatin dynamics in mouse zygotes
Source: Commun Biol. 2022 Jul 14;5:699. doi: 10.1038/s42003-022-03623-2 (PMC9283401; doi:10.1038/s42003-022-03623-2)
Supplement: Supplementary file 2 — Supplementary Information [file 42003_2022_3623_MOESM2_ESM.pdf]

Supplementary Information

Parental competition for the regulators of chromatin dynamics in mouse zygotes

Ooga et al

13 Supplementary Figures

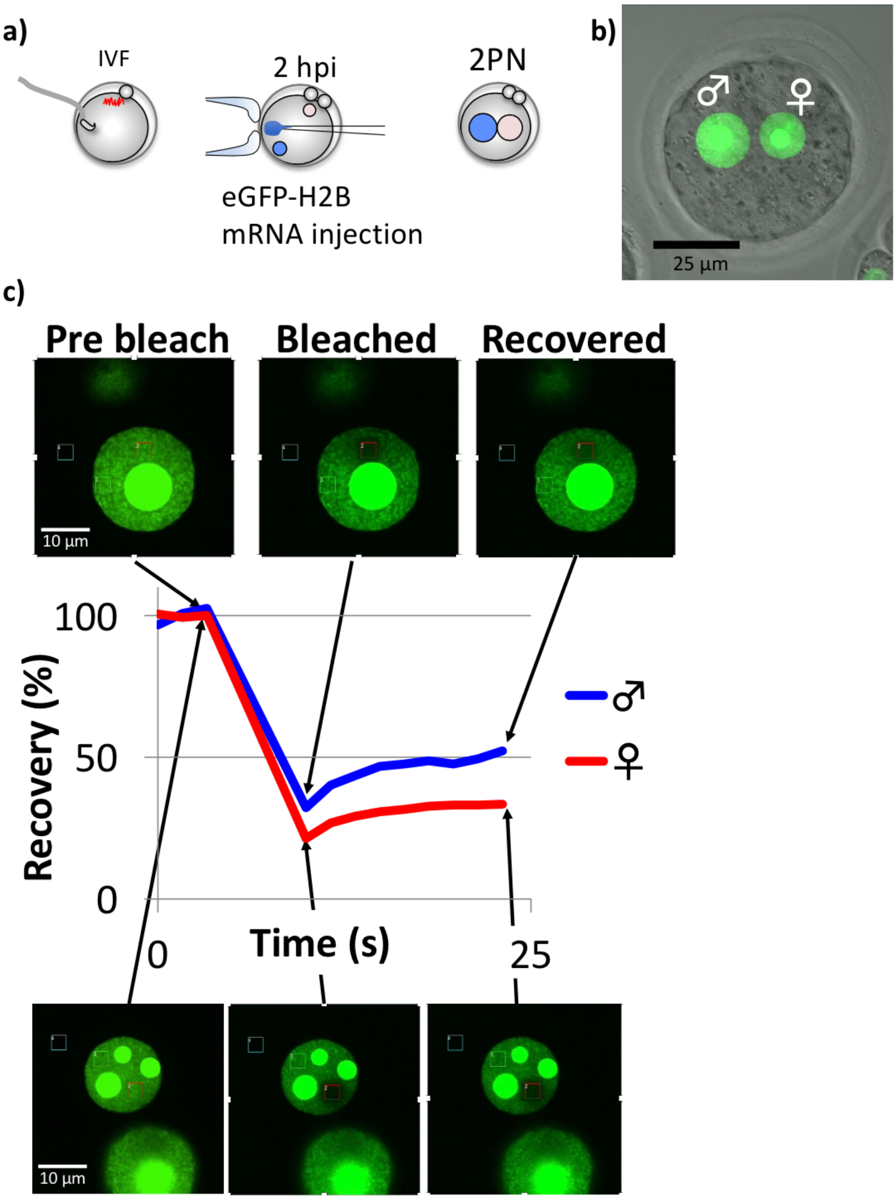

Supplementary Figure 1

zFRAP analysis

(**a**) In our previous study, IVF-derived zygotes were microinjected with mRNA at 2 hpi. (**b**) At 8 hpi, parental PNs were easily distinguished by size, as a male PN is larger than a female PN. Sufficient expression of eGFP-H2B was confirmed. (**c**) For zFRAP analysis, a specific region of interest (ROI) in the PNs was selected and bleached. Red rectangle indicates bleached ROI, green is reference, and light blue is background.

Compared to pre-bleaching, the fluorescence level drastically decreased after bleaching and then gradually recovered. Male PNs always exhibited greater fluorescence than the female PNs. Fluorescence levels at several points were plotted as a recovery curve.

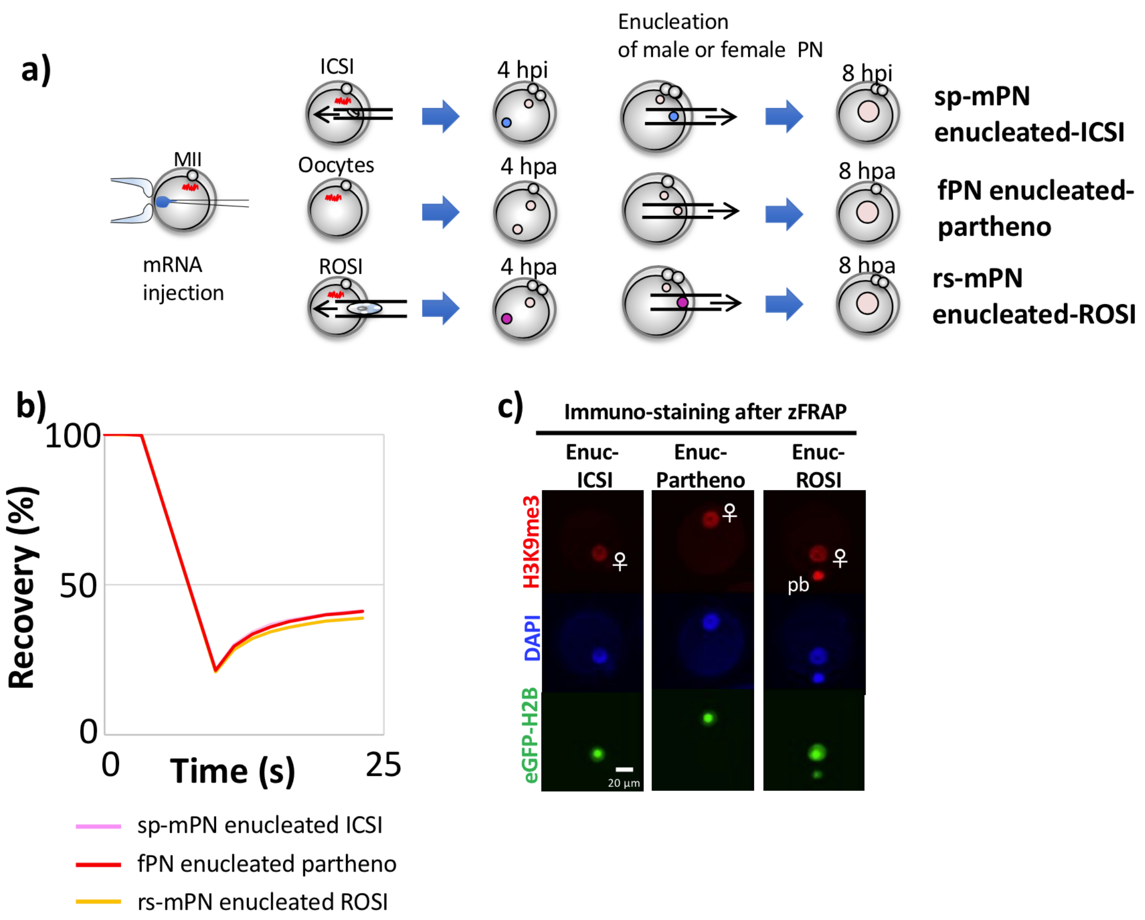

Supplementary Figure 2

Dependency of decreasing histone mobility of fPN on sp-mPN

(**a**) Illustration of the preparation of mPN-enucleated zygotes at 4 hpi (**b**) Recovery curve of mPN-enucleated partheno-zygotes. (**c**) In order to confirm correct PN selection, after zFRAP analysis, the zygotes were subjected immuno-staining of H3K9me3, as a fPN marker.

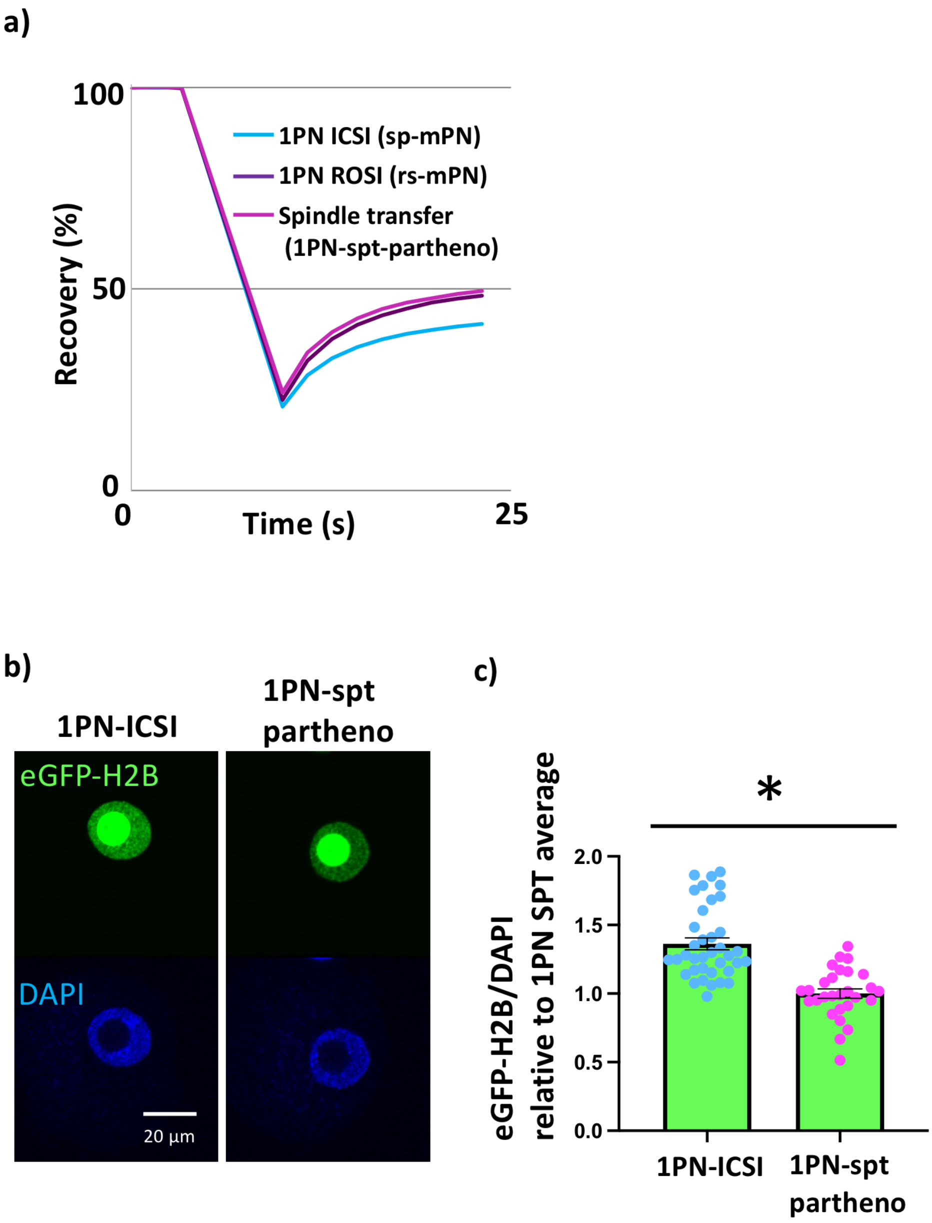

Supplementary Figure 3

Sperm-derived histone mobility decreasing ability functions to sp-mPN itself.

(a) Recovery curve of 1PN-zygotes. 1PN ICSI have only sp-mPN (sperm-derived mPN), 1PN ROSI have only rs-mPN (round spermatid-derived mPN), and 1PN-spt-partheno have only spt-fPN (spindle transfer-derived fPN). (b) eGFP expression level was examined in 1PN-ICSI and 1PN-spt-partheno zygotes. DNA was stained with DAPI. (c) The relative fluorescent signal of eGFP-H2B was plotted in the bar graph.

Error bars indicate SE. Asterisk indicate significant differences by unpaired t-test ($P<0.05$).

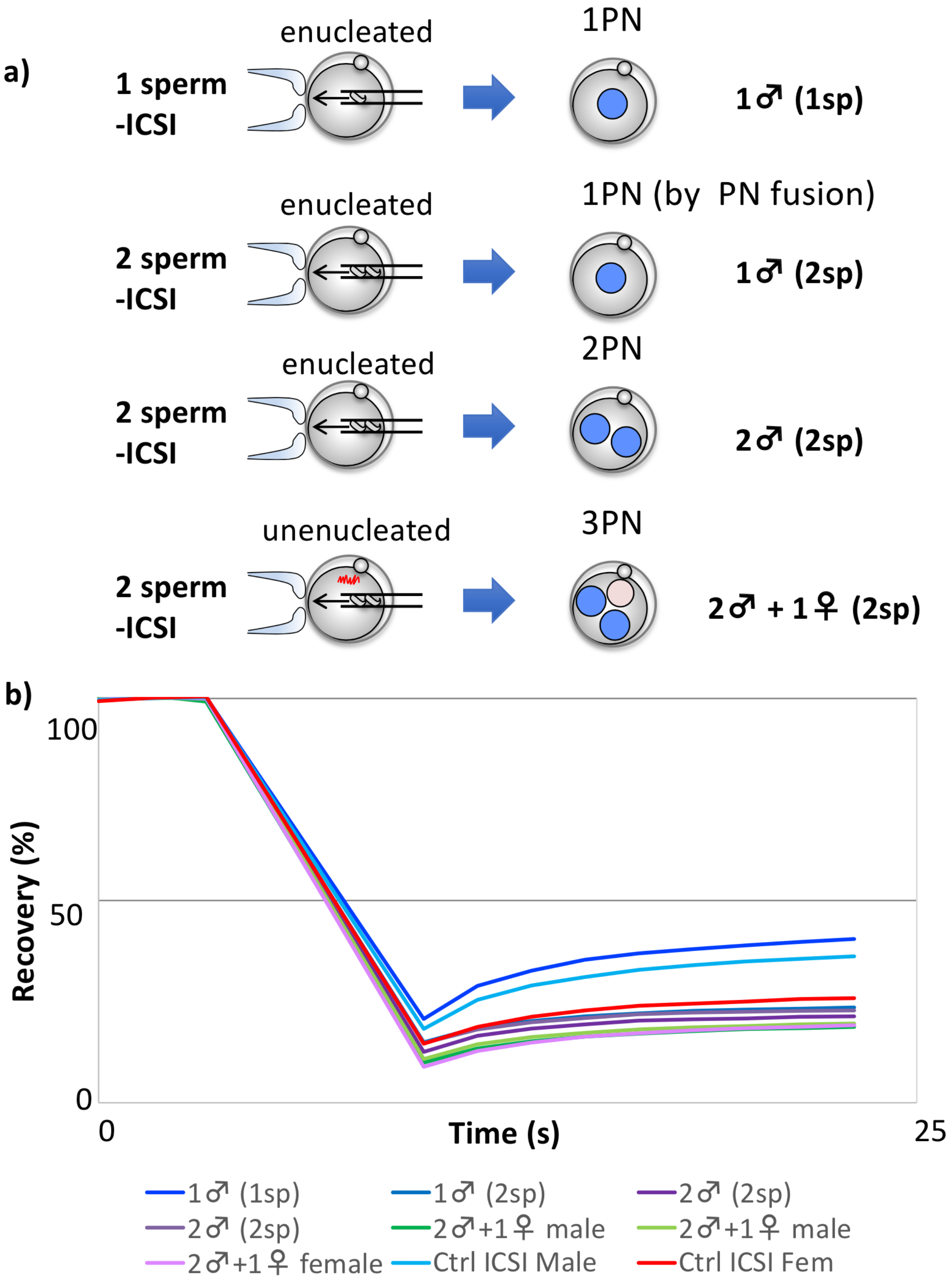

Supplementary Figure 4

Paternal repressive effect on histone mobility is dependent on the number of microinjected sperm

(**a**) Schematic illustration of the preparation of zygotes of 1♂ (1sp), 1♂ (2sp), 2♂ (2sp) and 2♂ + 1♀ (2sp). One or two sperm were microinjected into enucleated or un-enucleated MII oocytes. 1♂ (1sp) indicates that one sperm was injected, which resulted in the formation of a single sp-mPN. (**b**) Recovery curves of ICSI-zygotes microinjected with one or two sperm.

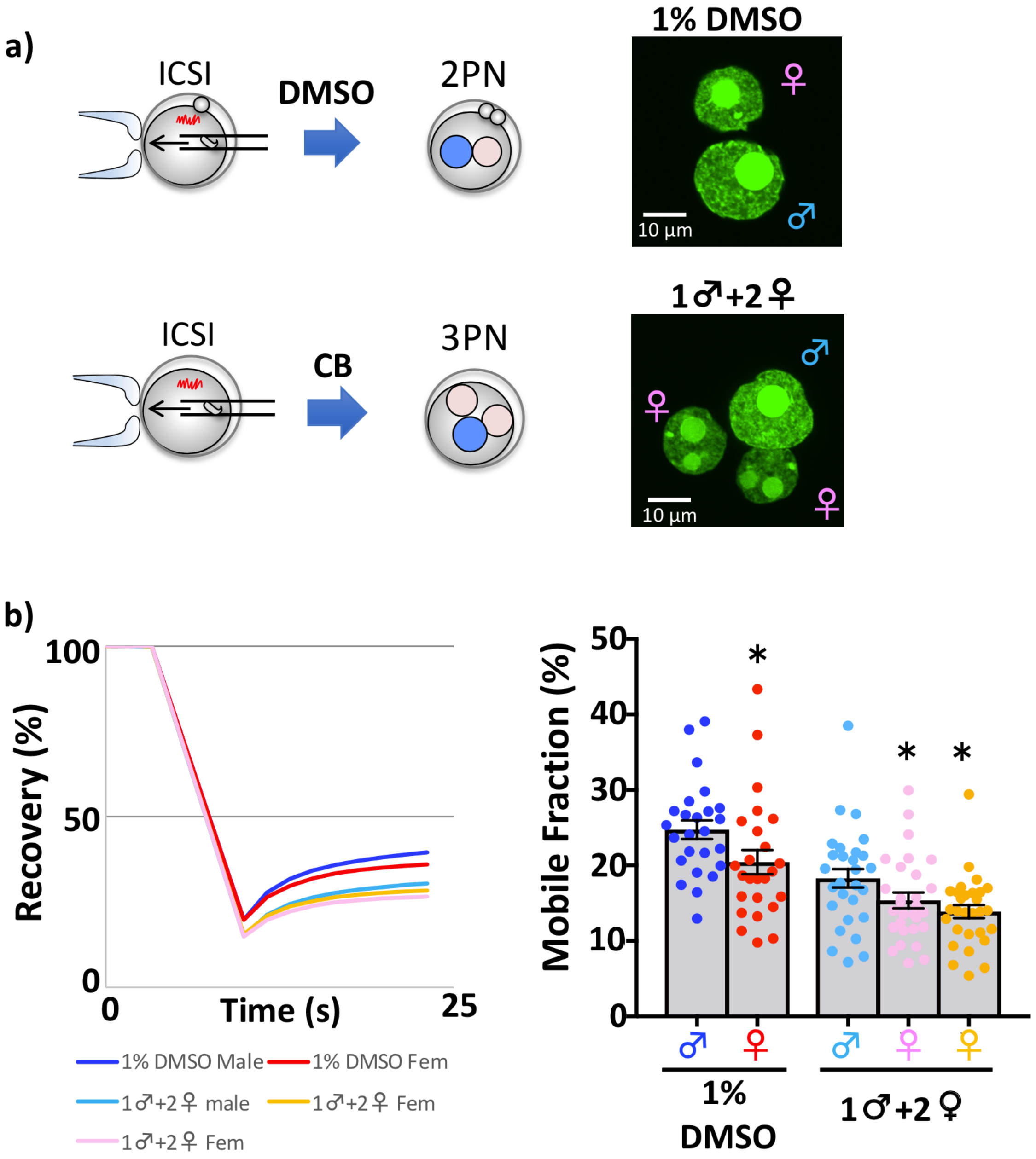

Supplementary Figure 5

Increases of fPN did not disrupt parental asymmetric pattern.

(**a**) Schematic illustration of the preparation of zygotes of 1♂ + 2♀. CB is an inhibitor of cytokinesis, which increases the fPN in the treated zygotes. Fluorescence images of the zygotes with 2 fPNs are shown. (**b**) Recovery curve and average MF scores of 1♂ + 2♀ and control zygotes are shown. Blue, red, light blue, pink, and orange dots are MF

scores of sp-mPN, fPN in control zygotes, and sp-mPN and two fPNs in CB treated-zygotes, respectively.

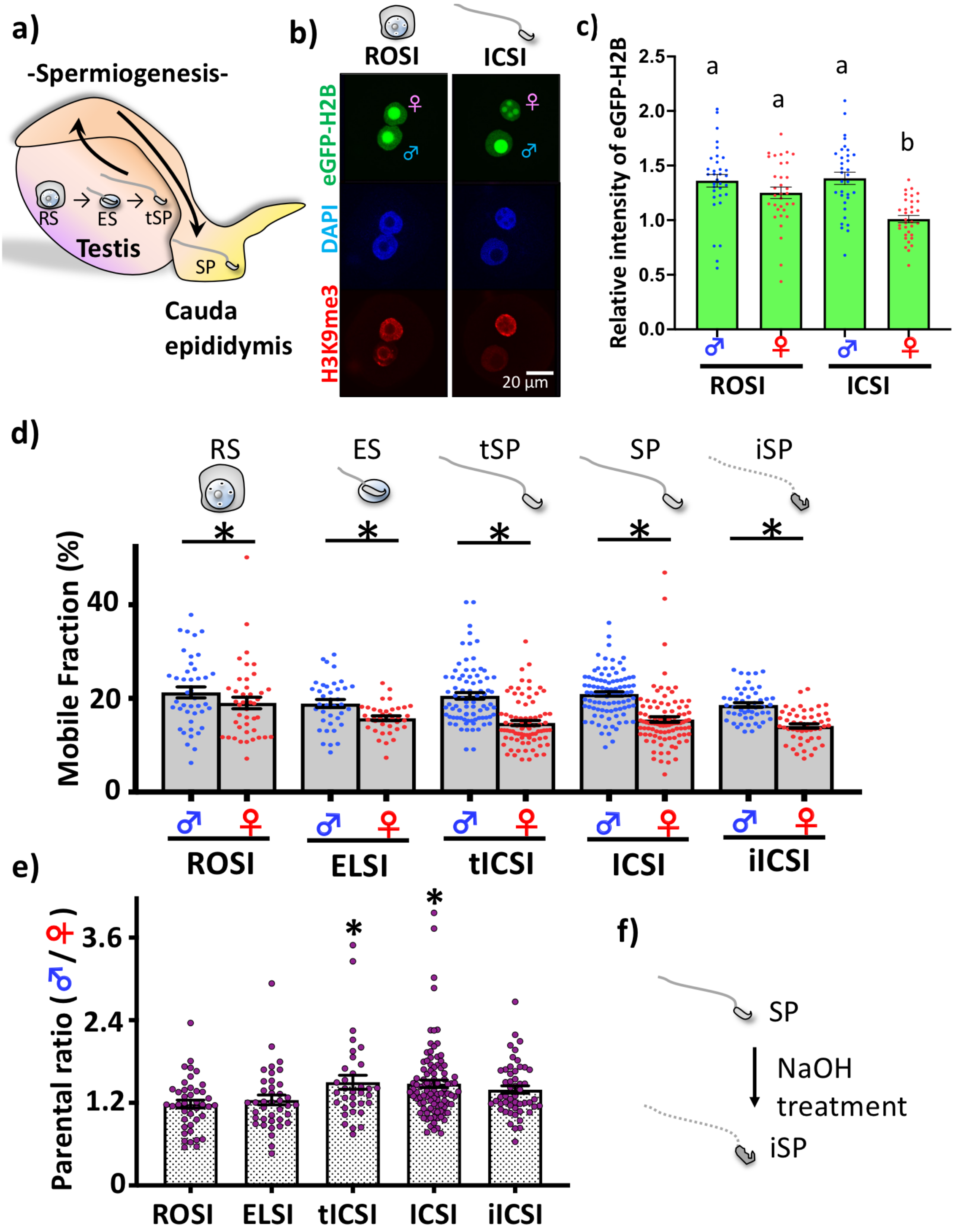

Supplementary Figure 6

Male germ cells acquired the ability to reduce histone mobility and resistance during spermiogenesis

(**a**) During spermiogenesis, which is the post-meiotic stage, male germ cells change the morphology dynamically and differentiate from round spermatid (RS) to elongating spermatid (ES) and testicular sperm (tSP) in the testis. After differentiation to tSP, they move to cauda epididymis from the testis and then mature to sperm (SP). Micro-insemination with this SP is called ICSI, and with round spermatid, elongating spermatid in the testis is as ROSI and ELSI (elongating spermatid injection), respectively. We referred to microinjection with immature tSP as testicular ICSI (tICSI). (**b**) The representative images of eGFP-H2B expressing ROSI-, and ICSI-zygotes were shown. (**c**) Relative expression levels of eGFP-H2B were shown in the bar graph. Error bars indicate the standard error. The different characters indicate significant differences by one-way ANOVA followed by Tukey’s multiple comparisons test. (**d**) zFRAP analysis with ROSI, ELSI, tICSI, ICSI, and ICSI with iSP (iICSI) was performed. The average MF scores of the zygotes are shown. Blue and red dots are the MF scores of the mPN and fPN, respectively. (**e**) Bar graph of the average MF score of the mPN/fPN ratio. Single purple dots indicate the ratio of single zygotes. The asterisks indicate significant differences as compared with ROSI by one-way ANOVA followed by Tukey’s multiple comparisons test. (**f**) By treating NaOH, SP loses its oocyte-activation capability (inactivated sperm: iSP).

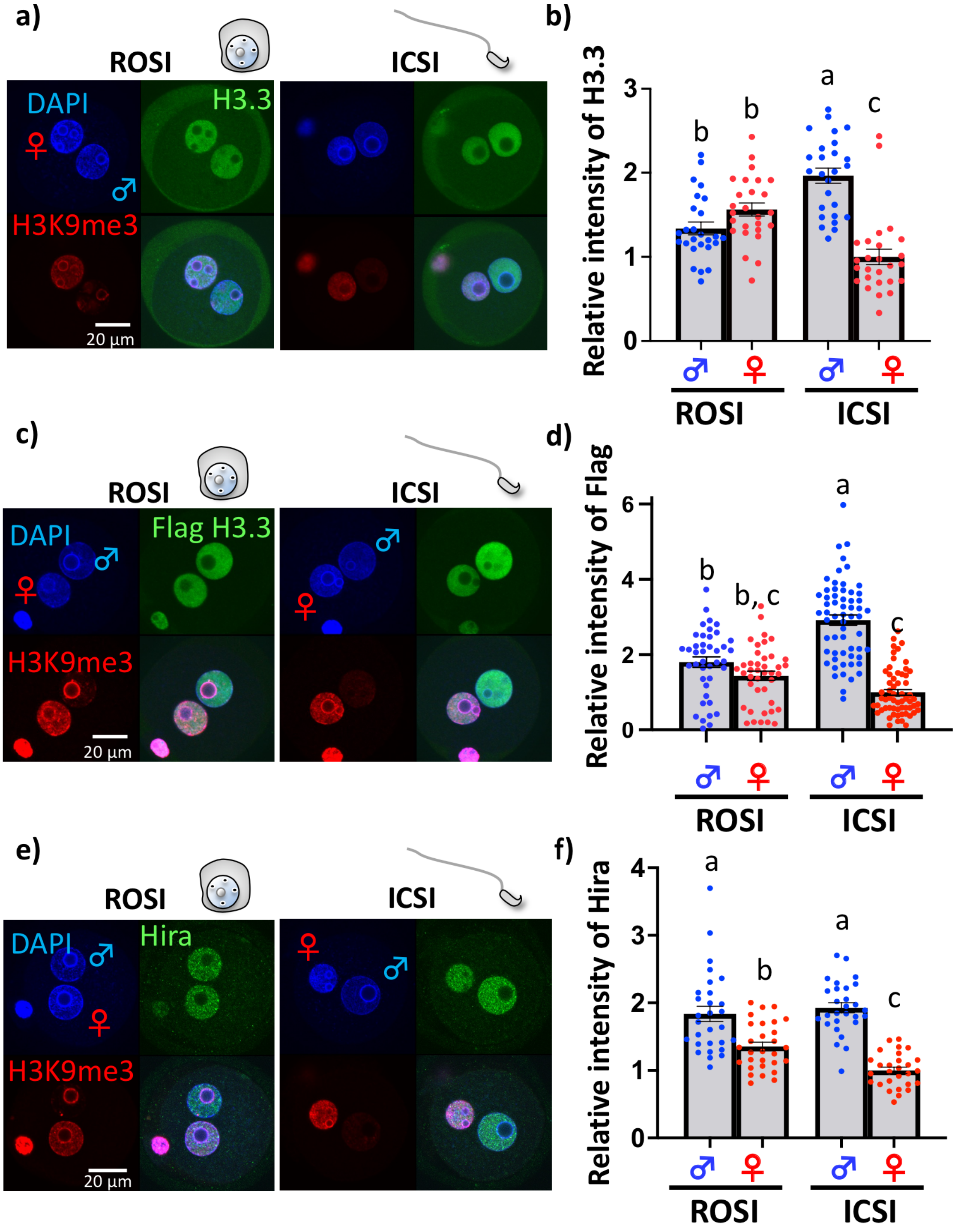

Supplementary Figure 7

The expression of H3.3 and its specific chaperon Hira in ROSI-, and ICSI-zygotes.

Immuno-staining analysis with using antibodies for H3.3 (a), Flag (b) and Hira (c) were performed. The representative images and the quantitative analysis data were shown.

All statistical analysis were performed by one-way ANOVA followed by Tukey’s multiple comparisons test. Different characters show significant differences. H3K9me3 (red) were used to distinguish the parental origin.

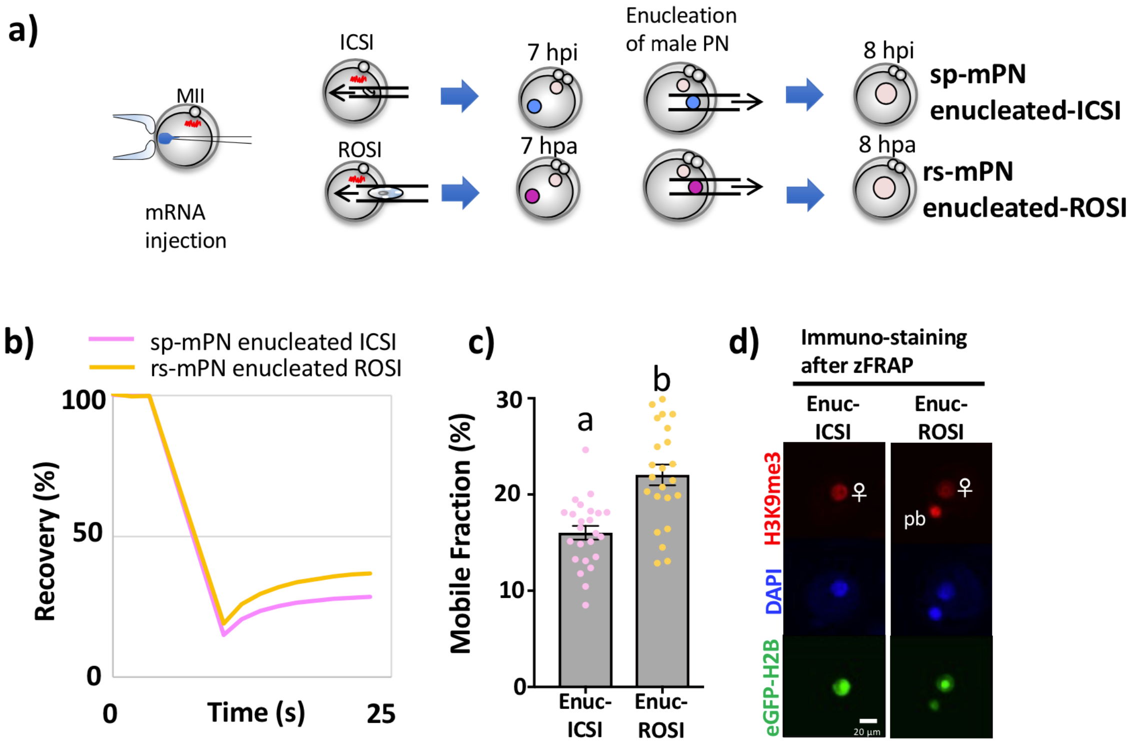

Supplementary Figure 8

Round spermatid did not harbor histone mobility decreasing ability

(**a**) Schematic illustration of the preparation of ICSI- and ROSI-zygotes without mPN.

(**b**) Recovery curve of sp-mPN enucleated ICSI- and rs-mPN enucleated ROSI-zygotes.

(**c**) The average MF scores are shown. Pink and orange dots indicate the MF score of each fPN of the zygotes. (**d**) Immuno-staining of H3K9me3 in zygotes after zFRAP analysis. H3K9me3 signals indicate correct discrimination of parental PN during the enucleation process.

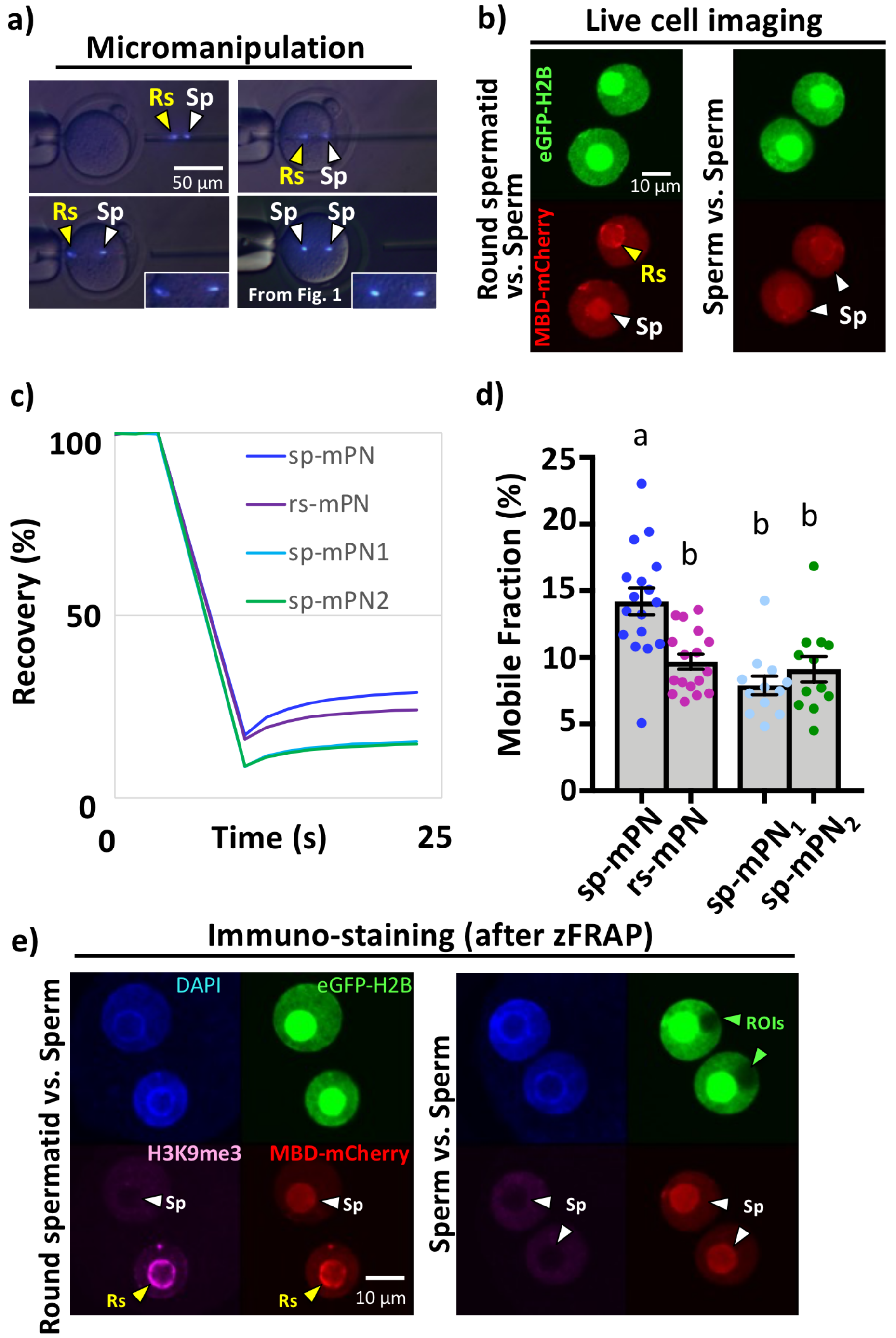

Supplementary Figure 9

Round spermatids were not resistant to the chromatin compaction effect of sperm

(**a**) Sperm head and round spermatid or sperm head and sperm head were co-injected into the enucleated oocytes at the same time. Yellow Rs and white Sp indicate round

spermatid and sperm, respectively. The insets are images at higher magnification. DNA was stained with Hoechst. (**b**) MBD-mCherry expression in the live zygotes during zFRAP analysis. Upper panel is eGFP-H2B expression. Lower red panel indicates MBD-mCherry expression. Triangle indicates the perinucleolar ring where MBD-mCherry was enriched in male PNs derived from round spermatid but not sperm. (**c**) Recovery curve indicating the average fluorescence recovery rate. sp-mPN, rs-mPN, sp-mPN#1, and sp-mPN#2 indicate the score of male PN derived from round spermatid and spermatozoa, and co-injected spermatozoon, respectively. (**d**) MF scores of 2PN androgenic zygotes are shown. Gray bar graph of average MF values. Single dots indicate the mobile fraction score obtained from either mPN derived from round spermatid or sperm. Different characters indicate significant differences ($p < 0.05$, by one-way ANOVA and Tukey's multi comparisons test). Error bar indicates the SE. (**e**) Immuno-staining analysis with antibody against H3K9me3. To confirm discrimination of the derivation of male PNs using MBD-mCherry, since the marker for male PN derived from round spermatid, H3K9me3, was stained. Purple indicates immuno-staining of H3K9me3, which only male PNs derived from round spermatid showed. Green triangle indicates the ROIs that were bleached during zFRAP analysis.

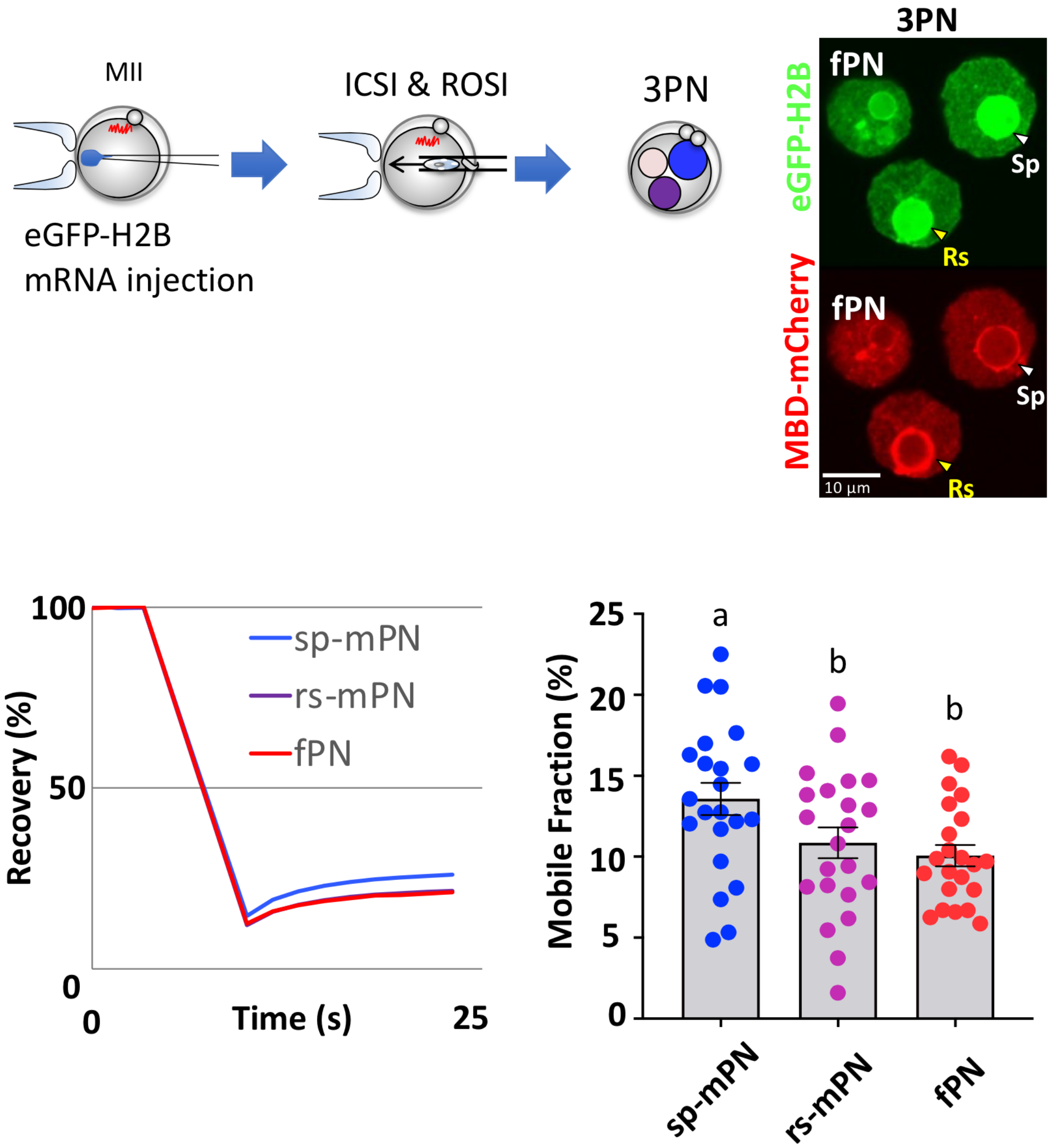

Supplementary Figure 10

Histone mobility in rs-mPN could be decreased to the same level as fPN

(**a**) Schematic illustration of the preparation of zygotes with sp-mPN, rs-mPN, and fPN. Sperm and round spermatids were co-injected into un-enucleated MII oocytes. (**b**) Fluorescence image of zygotes harboring the 3PN. MBD-mCherry showed preferential localization to the nucleolar ring of rs-mPN to distinguish the derivation of mPNs. (**c**), (**d**) Recovery curve and MF scores of sp-mPN, rs-mPN, and fPN in 3PN-zygotes.

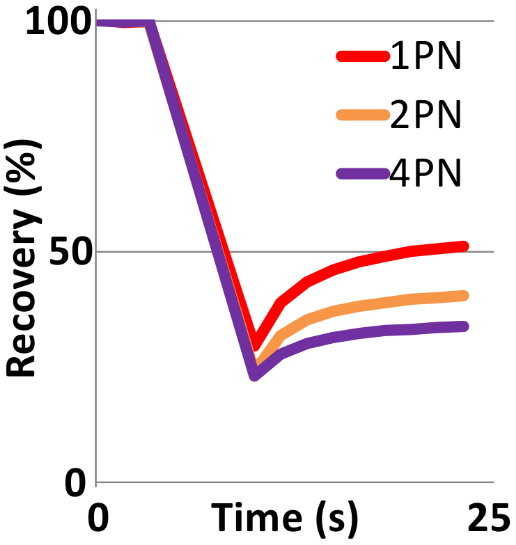

Supplementary Figure 11

Recovery curve of 1, 2, and 4PN parthenogenetic zygotes

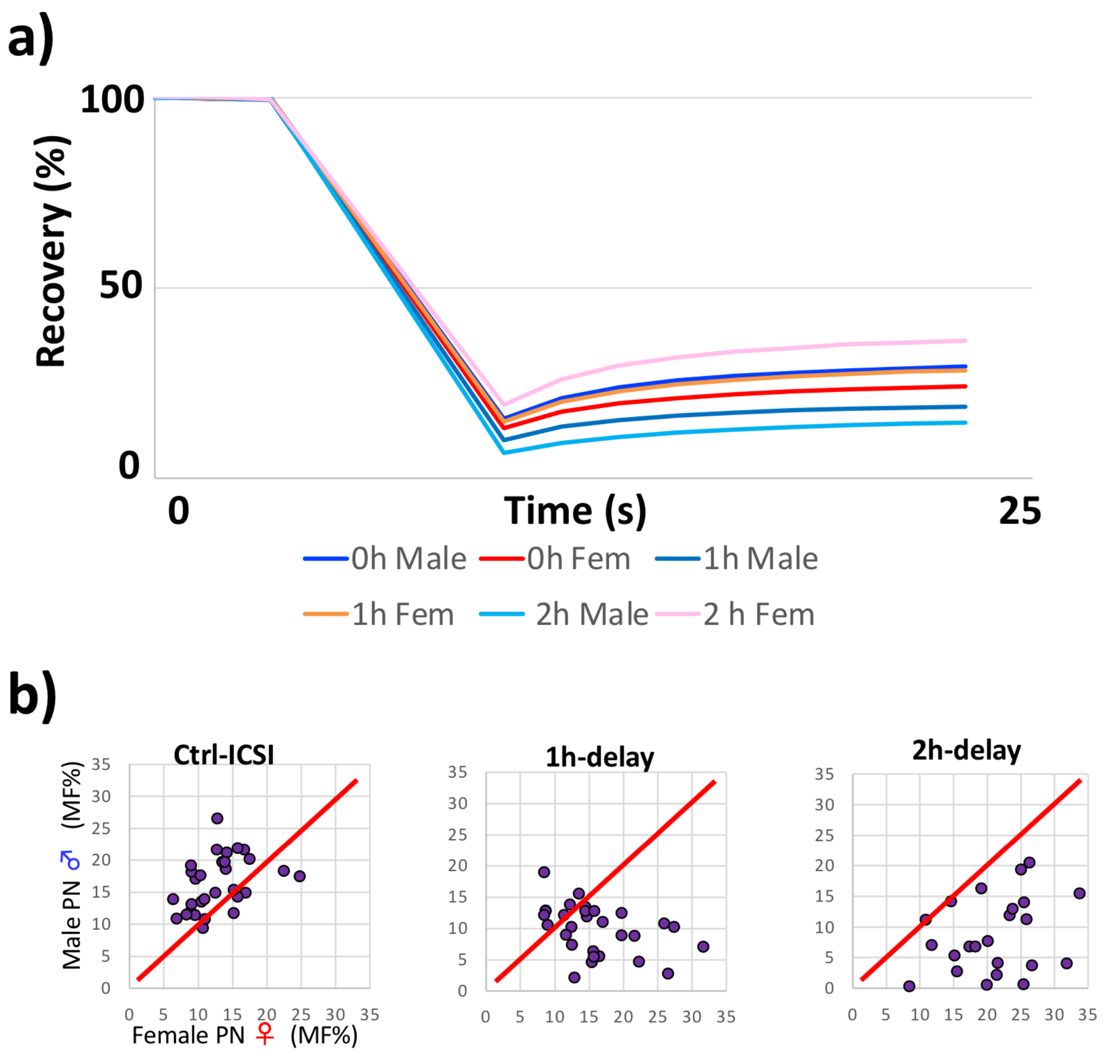

Supplementary Figure 12

Reversed parental asymmetric chromatin dynamics in delay ICSI-zygotes

(**a**) Recovery curve of control and 1, 2 h delay ICSI-zygotes.

(**b**) Purple single dots indicate MF scores of parental individual pronuclei. The Red line indicates the ♂ = ♀ borderline. Left side: ♂ > ♀, right side: ♂ < ♀.

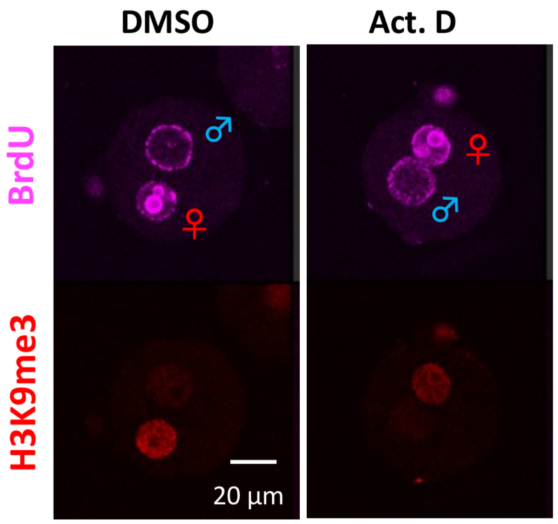

Supplementary Figure 13

Act D did not affect DNA polymerase activity

BrdU incorporation was visualized in IVF-derived zygotes with antibody against BrdU.

H3K9me3 was used to show the parental origin of PNs.
