# Supplementary material for: Parental competition for the regulators of chromatin dynamics in mouse zygotes
Source: Commun Biol. 2022 Jul 14;5:699. doi: 10.1038/s42003-022-03623-2 (PMC9283401; doi:10.1038/s42003-022-03623-2)
Supplement: Supplementary file 3 — Description of Additional Supplementary Files [file 42003_2022_3623_MOESM3_ESM.pdf]

Description of Additional Supplementary Files

File name: Supplementary Data 1

Description: The source data for generating the graphs and charts and sample numbers are shown in the main figures.

File name: Supplementary Data 2

Description: The results of statistical analysis for the main figures are shown.
