# Supplementary material for: Parental competition for the regulators of chromatin dynamics in mouse zygotes
Source: Commun Biol. 2022 Jul 14;5:699. doi: 10.1038/s42003-022-03623-2 (PMC9283401; doi:10.1038/s42003-022-03623-2)
Supplement: Supplementary file 6 — Reporting Summary [file 42003_2022_3623_MOESM6_ESM.pdf]

nature portfolio

Corresponding author(s): Masatoshi Ooga

Last updated by author(s): 2022/05/10

Reporting Summary

Nature Portfolio wishes to improve the reproducibility of the work that we publish. This form provides structure for consistency and transparency in reporting. For further information on Nature Portfolio policies, see our Editorial Policies and the Editorial Policy Checklist.

Statistics

For all statistical analyses, confirm that the following items are present in the figure legend, table legend, main text, or Methods section.

n/a	Confirmed	
☐	☒	The exact sample size (n) for each experimental group/condition, given as a discrete number and unit of measurement
☐	☒	A statement on whether measurements were taken from distinct samples or whether the same sample was measured repeatedly
☐	☒	The statistical test(s) used AND whether they are one- or two-sided<br>*Only common tests should be described solely by name; describe more complex techniques in the Methods section.*
☒	☐	A description of all covariates tested
☐	☒	A description of any assumptions or corrections, such as tests of normality and adjustment for multiple comparisons
☐	☒	A full description of the statistical parameters including central tendency (e.g. means) or other basic estimates (e.g. regression coefficient) AND variation (e.g. standard deviation) or associated estimates of uncertainty (e.g. confidence intervals)
☒	☐	For null hypothesis testing, the test statistic (e.g. F, t, r) with confidence intervals, effect sizes, degrees of freedom and P value noted<br>*Give P values as exact values whenever suitable.*
☒	☐	For Bayesian analysis, information on the choice of priors and Markov chain Monte Carlo settings
☒	☐	For hierarchical and complex designs, identification of the appropriate level for tests and full reporting of outcomes
☒	☐	Estimates of effect sizes (e.g. Cohen's d, Pearson's r), indicating how they were calculated

Our web collection on statistics for biologists contains articles on many of the points above.

Software and code

Policy information about availability of computer code

Data collection	FLUOVIEW ver 4.2 FV1200 and FV1000 (Olympus)
Data analysis	Image J (NIH), Excel (Microsoft), and Prism 9 (GraphPad)

For manuscripts utilizing custom algorithms or software that are central to the research but not yet described in published literature, software must be made available to editors and reviewers. We strongly encourage code deposition in a community repository (e.g. GitHub). See the Nature Portfolio guidelines for submitting code & software for further information.

Data

Policy information about availability of data

All manuscripts must include a data availability statement. This statement should provide the following information, where applicable:

- Accession codes, unique identifiers, or web links for publicly available datasets
- A description of any restrictions on data availability
- For clinical datasets or third party data, please ensure that the statement adheres to our policy

The datasets generated during and/or analyzed during the current study are available from the corresponding author on reasonable request.

Field-specific reporting

Please select the one below that is the best fit for your research. If you are not sure, read the appropriate sections before making your selection.

☒ Life sciences ☐ Behavioural & social sciences ☐ Ecological, evolutionary & environmental sciences

For a reference copy of the document with all sections, see nature.com/documents/nr-reporting-summary-flat.pdf

Life sciences study design

All studies must disclose on these points even when the disclosure is negative.

Sample size	No statistical method was used for pre-determine sample size.
Data exclusions	In some dot blot graphs of mobile fraction, a few dots, which showed extreme high scores, were not shown to avoid the graph becoming too small. But these scores were not eliminated to calculate the average score.
Replication	Similar tendency was obtained at least 3 times experiments was regarded as sufficient for the reproducibility.
Randomization	n/a
Blinding	n/a

Reporting for specific materials, systems and methods

We require information from authors about some types of materials, experimental systems and methods used in many studies. Here, indicate whether each material, system or method listed is relevant to your study. If you are not sure if a list item applies to your research, read the appropriate section before selecting a response.

Materials & experimental systems

n/a	Involved in the study	
☐	☒	Antibodies
☒	☐	Eukaryotic cell lines
☒	☐	Palaeontology and archaeology
☐	☒	Animals and other organisms
☒	☐	Human research participants
☒	☐	Clinical data
☒	☐	Dual use research of concern

Methods

n/a	Involved in the study	
☒	☐	ChIP-seq
☒	☐	Flow cytometry
☒	☐	MRI-based neuroimaging

Antibodies

Antibodies used

abcam Anti histone H3 (tri methyl K9) antibody ChIP Grade ab8898,
Cosmo Bio Anti H3.3 antibody Clone 4H2D7,
Merck Anti Flag M2 antibody F1804,
Active motif Anti-Hira #39558,

Validation

Validation statements of all antibodies are available on the manufacturers' websites:

H3K9me3
https://www.abcam.co.jp/histone-h3-tri-methyl-k9-antibody-chip-grade-ab8898/reviews/52119
This modification was used as marker for the discrimination of the derivation of parental genome.
In this study, this antibody was used for the discrimination and the results was definitely consistent with the a lot of previous studies.

H3.3
https://search.cosmobio.co.jp/cosmo_search_p/search_gate2/docs/CAC_/CE040B.20141021.pdf

Flag
https://www.sigmaaldrich.com/JP/ja/product/sigma/f1804?gclid=Cj0KCQjw3IqSBhCoARIsAMBkTb39qUKhSTHV18aFidslTFZA60Q2ihGn_klmREIR6hhbPVyePSHEvgEaAphtEALw_wcB

Hira
https://www.activemotif.com/catalog/details/39557/hira-antibody-mab-clone-wc119-2h11

Animals and other organisms

Policy information about studies involving animals; ARRIVE guidelines recommended for reporting animal research

Laboratory animals	Male: ICR adult mice, Female: B6D2F1 8 to 12-week-old mice
Wild animals	This study did not involve in wild animals.
Field-collected samples	This study did not involve the samples collected from the field.
Ethics oversight	All animal experiments were approved by the Ethics Committee of the University of Yamanashi (reference number: A29-24) and conducted in accordance with Guide for the Care and Use of Laboratory Animals and the ARRIVE guidelines.

Note that full information on the approval of the study protocol must also be provided in the manuscript.
